# Detection of Chikungunya Virus Circulation Using Sugar-Baited Traps during a Major Outbreak in French Guiana

**DOI:** 10.1371/journal.pntd.0004876

**Published:** 2016-09-08

**Authors:** Romain Girod, Amandine Guidez, Romuald Carinci, Jean Issaly, Pascal Gaborit, Emma Ferrero, Vanessa Ardillon, Albin Fontaine, Isabelle Dusfour, Sébastien Briolant

**Affiliations:** 1 Unité d’Entomologie Médicale, Institut Pasteur de la Guyane, Cayenne, French Guiana; 2 Cellule de l’Institut de Veille Sanitaire en Région Antilles-Guyane, Cayenne, French Guiana; 3 Groupe Interactions Virus-Insectes, Institut Pasteur, Paris, France; 4 Equipe Résidente de Recherche d’Infectiologie Tropicale, Division Expertise, Institut de Recherche Biomédicale des Armées, Brétigny-sur-Orge, France; 5 Unité de Recherche Associée 3012, Centre National de la Recherche Scientifique, Paris, France; 6 Institut de Recherche Biomédicale des Armées, Brétigny-sur-Orge, France; 7 Direction Interarmées du Service de Santé en Guyane, Cayenne, French Guiana; Independent Researcher, UNITED STATES

## Background and Objectives

Chikungunya virus (CHIKV) emerged in the Caribbean in November 2013 and rapidly spread through the Americas [1–3]. In French Guiana, transmission of the disease was highlighted for the first time in February 2014 [4]. It circulated in an epidemic form in the urban area of Cayenne until May 2015 [5]. Locally, the only recognized mosquito vector of CHIKV is *Aedes aegypti* [6]. As there is no specific treatment or vaccine against CHIKV, the best prevention strategy is to limit exposure to mosquitoes through individual and collective protection measures.

Vector control resources, both material and personnel, are limited in many countries. The early detection of virus transmission would help to implement adapted protective measures over time in areas where the risk of exposure is the highest. Such a strategy requires the availability of effective tools and methods to assess the risk of transmission in the field or even predict epidemic risk. Indeed, if epidemiological surveillance brings fundamental information to document a situation, available data are often incomplete and too late to effectively guide immediate vector control interventions.

The assessment of viral infection prevalence in mosquito populations is the most accurate way to estimate the risk of transmission to humans and to reveal viral circulation [7]. However, methods available to estimate infection rates require the collection of a large number of mosquitoes in the field, since percentage of infected ones is usually very low. This procedure is hardly compatible with the logistical capacity of most vector control operations.

Recent studies reported the development of trapping systems coupled with substrates impregnated with sugar on which mosquitoes come to feed and expel virus particles as an alternative method for the surveillance of virus transmission in the field [8–16]. This method relies on the use of Flinders Technology Associates (FTA) cards (Whatman), which inactivate viruses and preserve nucleic acids at ambient temperature, a prerequisite for further detection by molecular techniques.

In the present study, BG-sentinel traps (Biogents) [17–19] and ovitraps [20–22] were modified to passively collect viral RNA. The potential of these traps in the early detection of CHIKV was assessed in the urban area of Cayenne during the 2014–2015 outbreak.

## The Study

*Ae*. *aegypti* effective engorgement was first tested on honey-impregnated FTA cards over time in the laboratory. Equal feeding rates were observed at day 0 and day 7 after cards were impregnated with a honey solution at the concentration of 1.0 g/mL.

**Fig 1:**
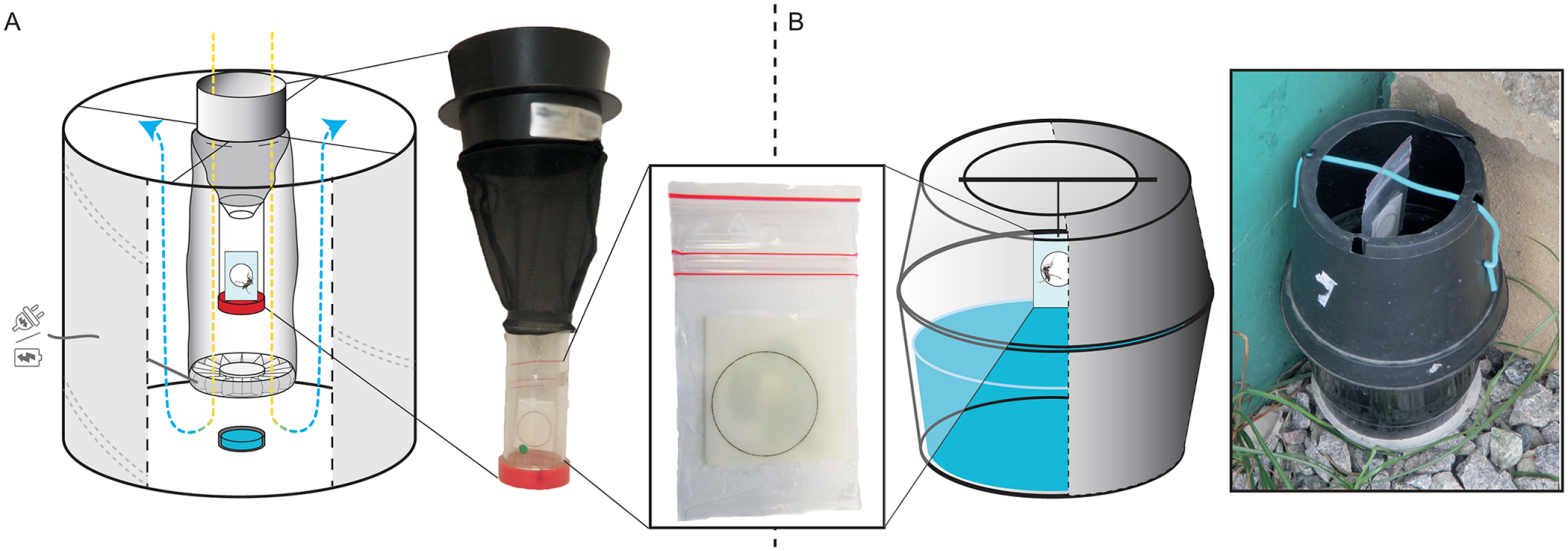
Photographs and schematic representations of traps. Photographs and schematic representations of (A) the modified BG-sentinel trap, showing the position of the tube containing the card into the trap, the incoming (yellow) and outgoing (blue) air flows, the blue tablet representing the attractant, and (B) the modified ovitrap, showing simple installation of the card.

BG-Sentinel traps were modified in order to hold an FTA card impregnated with honey solution on which mosquitoes could feed (Fig 1). The impregnated FTA card was placed in a small plastic bag pierced in the center so as to allow mosquitoes access to honey. The plastic bag with its card was placed in a tube which was closed at its bottom, before being hung on the collecting net of the trap. A funnel was placed at the top of the tube to capture all trapped mosquitoes. The BG-Lure (Biogents) was used as an attractant.

Ovitraps were adapted from the double sticky trap described by Chadee and Ritchie [22] in order to accommodate a card on which mosquitoes could feed (Fig 1). The attractant used was a maceration of one bean pod for 250 mL of water. No adhesive paper was used on the inner wall of the upper part of the trap, in order to not to interfere with the access and engorgement of females that are attracted but not held by the trap.

Both traps were installed and monitored in six different neighborhoods of the urban area of Cayenne. A total of 18 BG-Sentinel traps and 18 ovitraps were monitored weekly over a period ranging from November 27th, 2014, to March 4th, 2015.

A total of 446 usable FTA cards out of 468 installed cards (95.3%) and 17,729 mosquitoes, including 8,481 *Ae*. *aegypti* (47.8%), were collected during the field investigation. Alive and dead mosquitoes were separated upon return to the laboratory. *Ae*. *aegypti* females and males were then sorted and pooled with a maximum of 25 mosquitoes by capture site and sampling date. Plastic bags with FTA cards and mosquito pools were stored at –80°C before processing.

Extraction of RNA from cards was carried out either with the NucleoSpin RNA kit (Macherey-Nagel) or with the QIAamp Viral RNA Mini Kit (Qiagen). Extraction of RNA from mosquito pools was performed with TRIzol according to manufacturer's recommendations. Viral RNA was extracted from 219 cards recovered from modified ovitraps and 227 cards and 373 pools of *Ae*. *aegypti* females recovered from modified BG-Sentinel traps.

A real-time RT-PCR adapted from Panning et al. [23] was used to detect viral RNA in all RNA extracts.

No CHIKV RNA was detected on cards collected from BG-sentinel traps that were designed to house cards and collect mosquitoes. No CHIKV RNA was detected from the 3,665 *Ae*. *aegypti* females collected from the modified BG-sentinel traps, including 359 females that were alive at time of collection.

Two FTA cards collected from ovitraps were positive for CHIKV. The positive FTA cards had cycle threshold (Ct) scores of 34 and 37, whilst a CHIKV-positive control, extracted from virus culture supernatant, had a Ct of 18. The presence of CHIKV RNA on the two ovitrap samples was confirmed by end-point RT-PCR adapted from Naze et al. [24]. Then they were Sanger sequenced (Macrogen Company). The obtained nucleotide sequences of the sample with a Ct of 34 showed 100% of identity with a Caribbean CHIKV strain (GenBank: LN898112) when aligned with MEGA5.2 (Informer Technologies Inc,). No exploitable nucleotide sequence was obtained with the other sample.

The proposed modified ovitrap is convenient because of its simple design and independence to energy. The average percentage of usable FTA cards after a week of exposure in the field was high with this trap (93.6%), even if less than with modified BG-sentinel traps (97.0%). On several occasions, the cards could not be recovered or had degraded through consumption and destruction by other insects, such as cockroaches and ants.

The relative sensitivity of virus RNA detection in honey-impregnated FTA cards cannot be easily determined in the field without monitoring the contact between infectious mosquitoes and the cards. The ovitrap used was not designed to collect mosquitoes, and females were free to leave after laying eggs. Thus, no information on the species that secreted saliva in the card was available. Anyway, we showed that providing an easy access to sugar to infectious females attracted to lay eggs could be sufficient to capture viral RNA. Because RNA can be preserved for several days in the field, these traps can be seen as valuable tools to easily screen for virus transmission on a large geographic scale. This strategy can limit the time required to sort and analyze mosquitoes, especially when mosquitoes are in great number and mosquitoes’ infection rates are low. Absence of CHIKV RNA in cards collected on BG-Sentinel can result from an absence of infectious mosquitoes that have been trapped, and it is thereby not possible to draw any conclusion about a lower sensitivity of BG-Sentinel as compared to ovitraps during the current study.

## Concluding Remarks and Perspectives

Current methods available to detect the circulation of arboviruses (and thus CHIKV) in mosquito populations are labor intensive and difficult to implement by vector control operators, particularly in isolated places. In this study, CHIKV RNA was detected on honey-impregnated FTA cards installed in simple and inexpensive ovitraps, suggesting that early detection of CHIKV expelled by mosquitoes during their sugar meal presents a promising alternative for surveillance of mosquito-borne arboviral diseases.

Complementary work should be done in the laboratory in order to assess the sensitivity of such traps for the early detection of CHIKV in *Ae*. *aegypti* populations. In particular, CHIKV degradation over time on impregnated cards should be assessed in order to determine the maximum time interval at which traps should be checked in the field. In addition, the minimal number of infected mosquitoes taking a sugar meal and allowing detection of viral RNA on the FTA card should be explored to give indication on the sensitivity of the method. Moreover, it is not possible at this stage to determine if viral RNA came from mosquito saliva and/or excreta. Further experimental work in the laboratory is needed to address these points [25].

The use of modified ovitraps in this study allowed for confirmation of only one FTA card out of 234 placed in the field (0.43%) as positive for the presence of CHIKV RNA. It still represents a low yield for a time-consuming and laborious work, even if clearly less demanding than processing mosquitoes. It could be pointed out that human surveillance should be more efficient than looking for rare positive FTA cards during an epidemic involving arboviral diseases causing a high prevalence of symptomatic human cases (e.g., chikungunya). However, the use of honey-impregnated FTA cards installed in ovitraps could be adapted to detect viruses with a zoonotic transmission cycle involving humans as an incidental host, such as West Nile virus or Saint Louis encephalitis virus. In addition, this method can be valuable to detect circulation of viruses in locations where no epidemiological data are available or for arboviruses that produce a high percentage of asymptomatic cases, such as Zika virus, whose circulation has been demonstrated recently in South America [26]. The method could also be applied to early detection of the circulation of parasites responsible for malaria or other pathogens transmitted by mosquitoes and other insect vectors [27].
